# Supplementary material for: Acute MPTP Treatment Impairs Dendritic Spine Density in the Mouse Hippocampus
Source: Brain Sci. 2021 Jun 23;11(7):833. doi: 10.3390/brainsci11070833 (PMC8301854; doi:10.3390/brainsci11070833)
Supplement: Supplementary file 1 [file brainsci-11-00833-s001.zip › brainsci-1230745-supplementary.pdf]

brain sciences

MDPI

Supplementary Materials

Acute MPTP treatment alters neural architecture in the mouse hippocampus

Poornima D. E. Weerasinghe-Mudiyanselage[1,†], Mary Jasmin Ang[1,2,†], Mai Wada[1], Sung-Ho Kim[1], Taekyun Shin[3], Miyoung Yang[4,*], Changjong Moon[1,*]

1 Department of Veterinary Anatomy, College of Veterinary Medicine and BK21 FOUR Program, Chonnam National University, Gwangju 61186, South Korea; 208314@jnu.ac.kr (P.D.E.W.M.)); wataametokatatu-muri@gmail.com (M.W.); shokim@chonnam.ac.kr (S-H.K.); moonc@chonnam.ac.kr (C.M.)
2 College of Veterinary Medicine, University of the Philippines Los Baños, Los Baños 4031, Philippines; mcang3@up.edu.ph (M.J.A.)
3 Department of Veterinary Anatomy, College of Veterinary Medicine, Jeju National University, Jeju 63243, South Korea; shint@jejunu.ac.kr (T.S.)
4 Department of Anatomy, Wonkwang University School of Medicine, Jeonbuk 54538, South Korea; yangm@wku.ac.kr (M.Y.)
* Correspondence: yangm@wku.ac.kr; Tel.: +82-63-850-6758 (M.Y.), moonc@chonnam.ac.kr; Tel.: +82-62-530-2838 (C.M.)
† The first two authors (P.D.E.W-M and M.J.A.) contributed equally to this work.

Supplementary Table S1–S3

To test the main effects of treatment and time (or distance), as well as their interactions with results of TH immunoreactivities, behavioral test, and Sholl analyses, two-way ANOVA followed by Šidák's multiple comparison test was used. The results of the ANOVAs are presented in supplementary Tables S1–S3.

Table S1. Two-way ANOVA of the effects of treatment and time on motor function (measured using the rotarod test) and dopaminergic cell loss (measured using TH immunostaining) in mice.

	Figure	Treatment[1]	Time[2]	Interaction
Rotarod test				
Rotation speed attained	Fig. 1A	F(1, 14) = 8.587<br>p = 0.0110	F(3, 42) = 16.08<br>p < 0.0001	F(3, 42) = 7.179<br>p = 0.0005
Latency to fall	Fig. 1A	F(1, 14) = 7.308<br>p = 0.0171	F(3, 42) = 13.32<br>p < 0.0001	F(3, 42) = 7.650<br>p = 0.0003
TH immunoreactivity				
No. of TH-positive cells in the substantia nigra	Fig. 1B	F(1, 10) = 61.08<br>p < 0.0001	F(2, 20) = 2.276<br>p = 0.1287	F(2, 20) = 0.02257<br>p = 0.9777

[1]Acute treatment of vehicle (saline) or MPTP (22 mg/kg) were given four times with 2 h intervals within a single day.
[2]At 1, 2, 4 and 8 days post-treatment, rotarod tests were performed, and at 1, 8 and 16 days post-treatment, TH expressions in the substantia nigra of brains were analyzed in vehicle- or MPTP-treated mice.
Abbreviations: ANOVA, analysis of variance; MPTP, 1-methyl-4-phenyl-1,2,3,6-tetrahydropyridine; OD, optical density; TH, tyrosine hydroxylase.

Table S2. Two-way ANOVA of the effects of treatment and distance on dendritic arborization (measured by Sholl analysis) in the mouse hippocampus.

	Figure	Treatment[1]	Distance[2]	Interaction
1 day post-treatment				
CA1 apical	Fig. 2B	$F(1, 10) = 0.9379$<br>$p = 0.3557$	$F(22, 220) = 71.31$<br>$P<0.0001$	$F(22, 220) = 0.3581$<br>$p = 0.9969$
CA1 basal	Fig. 2B	$F(1, 10) = 0.4732$<br>$p = 0.5072$	$F(11, 110) = 228.0$<br>$p < 0.0001$	$F(11, 110) = 1.117$<br>$p = 0.3548$
DG	Fig. 4B	$F(1, 10) = 0.4218$<br>$p = 0.5307$	$F(16, 160) = 178.8$<br>$p < 0.0001$	$F(16, 160) = 0.5895$<br>$p = 0.8886$
8 days post-treatment				
CA1 apical	Fig. 2B	$F(1, 10) = 1.049$<br>$p = 0.3298$	$F(22, 220) = 148.5$<br>$p < 0.0001$	$F(22, 220) = 0.5709$<br>$p = 0.9396$
CA1 basal	Fig. 2B	$F(1, 10) = 0.8124$<br>$p = 0.3886$	$F(11, 110) = 130.6$<br>$p < 0.0001$	$F(11, 110) = 0.2423$<br>$p = 0.9937$
DG	Fig. 4B	$F(1, 10) = 0.01893$<br>$p = 0.8933$	$F(16, 160) = 172.6$<br>$p < 0.0001$	$F(16, 160) = 0.9369$<br>$p = 0.5286$
16 days post-treatment				
CA1 apical	Fig. 2B	$F(1, 10) = 2.597$<br>$p = 0.1381$	$F(22, 220) = 148.6$<br>$p < 0.0001$	$F(22, 220) = 1.253$<br>$p = 0.2062$
CA1 basal	Fig. 2B	$F(1, 10) = 0.2920$<br>$p = 0.6007$	$F(11, 110) = 183.6$<br>$p < 0.0001$	$F(11, 110) = 1.800$<br>$p = 0.0623$
DG	Fig. 4B	$F(1, 10) = 4.726$<br>$p = 0.0548$	$F(16, 160) = 192.1$<br>$p < 0.0001$	$F(16, 160) = 0.9521$<br>$p = 0.5117$

[1]Acute treatment of vehicle (saline) and MPTP (22 mg/kg) were given four times with 2 h intervals within a single day.
[2]At different radial distance from neuronal soma, the number of dendritic intersections was counted in each hippocampal subregion, using Sholl analysis.
Abbreviations: ANOVA, analysis of variance; CA1, *cornu ammonis* 1; DG, dentate gyrus; MPTP, 1-methyl-4-phenyl-1,2,3,6-tetrahydropyridine.

Table S3. Two-way ANOVA of the effects of treatment and time on total dendritic length, dendritic branch points, and spine density in the mouse hippocampus.

	Figure	Treatment[1]	Time[2]	Interaction
Total dendritic length				
CA1 apical	Fig. 3A	F (1, 10) = 1.268<br>p = 0.2865	F (2, 20) = 1.872<br>p = 0.1797	F (2, 20) = 2.250<br>p = 0.1315
CA1 basal	Fig. 3A	F (1, 10) = 1.027<br>p = 0.3347	F (2, 20) = 0.8121<br>p = 0.4580	F (2, 20) = 0.4502<br>p = 0.6438
DG	Fig. 4C	F (1, 10) = 0.6776<br>p = 0.4296	F (2, 20) = 4.915<br>p = 0.0184	F (2, 20) = 1.303<br>p = 0.2937
Dendritic branch points				
CA1 apical	Fig. 3B	F (1, 10) = 1.544<br>p = 0.2424	F (2, 20) = 3.549<br>p = 0.0480	F (2, 20) = 3.883<br>p = 0.0376
CA1 basal	Fig. 3B	F (1, 10) = 1.580<br>p = 0.2374	F (2, 20) = 0.4935<br>p = 0.6177	F (2, 20) = 0.4462<br>p = 0.6463
DG	Fig. 4C	F (1, 10) = 7.019<br>p = 0.0243	F (2, 20) = 1.800<br>p = 0.1911	F (2, 20) = 1.600<br>p = 0.2267
Spine density				
CA1 apical	Fig. 5A	F (1, 10) = 8.748<br>p = 0.0143	F (2, 20) = 5.094<br>p = 0.0163	F (2, 20) = 1.655<br>p = 0.2161
CA1 basal	Fig. 5B	F (1, 10) = 11.09<br>p = 0.0076	F (2, 20) = 11.01<br>p = 0.0006	F (2, 20) = 0.7967<br>p = 0.4646
DG	Fig. 5C	F (1, 10) = 11.02<br>p = 0.0077	F (2, 20) = 11.80<br>p = 0.0004	F (2, 20) = 0.9540<br>p = 0.4020

[1]Acute treatment of vehicle (saline) or MPTP (22 mg/kg) were given four times with 2 h intervals within a single day.
[2]At 1, 8 and 16 days post-treatment, total dendritic length, dendritic branch points per neuron and spine density per 10 μm dendrite in Golgi-impregnated hippocampal neurons were analyzed in vehicle- or MPTP-treated mice.
Abbreviations: ANOVA, analysis of variance; CA1, *cornu ammonis* 1; DG, dentate gyrus; MPTP, 1-methyl-4-phenyl-1,2,3,6-tetrahydropyridine.
